# A matching-adjusted indirect comparison of combination nivolumab plus ipilimumab with BRAF plus MEK inhibitors for the treatment of *BRAF*-mutant advanced melanoma^☆^

**DOI:** 10.1016/j.esmoop.2021.100050

**Published:** 2021-02-06

**Authors:** A.A. Tarhini, K. Toor, K. Chan, D.F. McDermott, P. Mohr, J. Larkin, F.S. Hodi, C.-H. Lee, J.I. Rizzo, H. Johnson, A. Moshyk, S. Rao, S. Kotapati, M.B. Atkins

**Affiliations:** 1 Departments of Cutaneous Oncology and Immunology, H. Lee Moffitt Cancer Center and Research Institute, Tampa, USA; 2 Evidence Synthesis and Decision Modeling, Precision HEOR, Vancouver, Canada; 3 Medical Oncology, Beth Israel Deaconess Medical Center and Harvard Medical School, Boston, USA; 4 Department of Dermatology, Elbe Kliniken Buxtehude, Buxtehude, Germany; 5 Medical Oncology, The Royal Marsden Hospital, London, UK; 6 Medical Oncology, Dana-Farber/Harvard Cancer Center, Boston, USA; 7 US Health Economics and Outcome Research, Metastatic Melanoma, Bristol Myers Squibb, Princeton, USA; 8 Oncology Clinical Development, Bristol Myers Squibb, Princeton, USA; 9 Worldwide Health Economics and Outcomes Research, Melanoma, Bristol Myers Squibb, Uxbridge, UK; 10 Worldwide Health Economics and Outcomes Research, Melanoma, Bristol Myers Squibb, Princeton, USA; 11 Worldwide Medical, Melanoma, Bristol Myers Squibb, Princeton, USA; 12 Medical Oncology, Georgetown Lombardi Comprehensive Cancer Center, Washington, DC, USA

**Keywords:** advanced melanoma, BRAF/MEK inhibitors, ipilimumab, matching-adjusted indirect comparison, nivolumab

## Abstract

**Background:**

Approved first-line treatments for patients with *BRAF* V600–mutant advanced melanoma include nivolumab (a programmed cell death protein 1 inhibitor) plus ipilimumab (a cytotoxic T lymphocyte antigen-4 inhibitor; NIVO+IPI) and the BRAF/MEK inhibitors dabrafenib plus trametinib (DAB+TRAM), encorafenib plus binimetinib (ENCO+BINI), and vemurafenib plus cobimetinib (VEM+COBI). Results from prospective randomized clinical trials (RCTs) comparing these treatments have not yet been reported. This analysis evaluated the relative efficacy and safety of NIVO+IPI versus DAB+TRAM, ENCO+BINI, and VEM+COBI in patients with *BRAF*-mutant advanced melanoma using a matching-adjusted indirect comparison (MAIC).

**Patients and methods:**

A systematic literature review identified RCTs for DAB+TRAM, ENCO+BINI, and VEM+COBI in patients with *BRAF*-mutant advanced melanoma. Individual patient-level data for NIVO+IPI were derived from the phase III CheckMate 067 trial (*BRAF*-mutant cohort) and restricted to match the inclusion/exclusion criteria of the comparator trials. Treatment effects for overall survival (OS) and progression-free survival (PFS) were estimated using Cox proportional hazards and time-varying hazard ratio (HR) models. Safety outcomes (grade 3 or 4 treatment-related adverse events) with NIVO+IPI and the comparators were compared.

**Results:**

In the Cox proportional hazards analysis, NIVO+IPI showed improved OS compared with DAB+TRAM (HR = 0.53; 95% confidence interval [CI], 0.39-0.73), ENCO+BINI (HR = 0.60; CI, 0.42-0.85), and VEM+COBI (HR = 0.50; CI, 0.36-0.70) for the overall study period. In the time-varying analysis, NIVO+IPI was associated with significant improvements in OS and PFS compared with the BRAF/MEK inhibitors 12 months after treatment initiation. There were no significant differences between NIVO+IPI and BRAF/MEK inhibitor treatment from 0 to 12 months. Safety outcomes favored DAB+TRAM over NIVO+IPI, whereas NIVO+IPI was comparable to VEM+COBI.

**Conclusion:**

Results of this MAIC demonstrated durable OS and PFS benefits for patients with *BRAF*-mutant advanced melanoma treated with NIVO+IPI compared with BRAF/MEK inhibitors, with the greatest benefits noted after 12 months.

## Highlights

• First-line treatments for *BRAF* V600-mutant melanoma include NIVO+IPI and BRAF/MEK inhibitors.
• Results from prospective RCTs comparing NIVO+IPI and BRAF/MEK inhibitors have not yet been reported.
• This MAIC evaluated NIVO+IPI versus BRAF/MEK inhibitors for *BRAF*-mutant advanced melanoma.
• OS and PFS benefits were noted with NIVO+IPI versus BRAF/MEK inhibitors beginning at 12 months.
• These findings may provide information relevant to the selection of treatments for *BRAF*-mutant advanced melanoma.

## Introduction

Patients with unresectable advanced (stage III/IV) melanoma have historically had limited treatment options and faced a poor prognosis [median overall survival (OS) with metastatic disease of <12 months].^1^^,^^2^ However, several treatments have been introduced over the past decade that have dramatically improved outcomes in this setting, especially for patients with *BRAF* V600–mutant disease, who constitute approximately 50% of the metastatic cutaneous melanoma population.^3^ First-line treatments approved by the US Food and Drug Administration (FDA) for patients with *BRAF*-mutant melanoma include—among others—the immunotherapy combination of nivolumab [a programmed death (PD)-1 inhibitor] plus ipilimumab [a cytotoxic T-lymphocyte antigen-4 (CTLA-4) inhibitor; NIVO+IPI] and targeted therapy combinations with the BRAF/MEK inhibitors dabrafenib plus trametinib (DAB+TRAM), encorafenib plus binimetinib (ENCO+BINI), and vemurafenib plus cobimetinib (VEM+COBI).^4^ In the phase III CheckMate 067 study (NCT01844505), NIVO+IPI or NIVO alone was associated with significant and durable OS benefits compared with IPI alone in previously untreated patients with advanced melanoma at 5-year follow-up, regardless of *BRAF*-mutation status.^5^^,^^6^ In the subgroup of patients treated with NIVO+IPI having tumors harboring *BRAF* mutations, the 5-year OS rate was 60%.^6^ BRAF/MEK inhibitor combinations have also significantly improved long-term OS in previously untreated patients with *BRAF*-mutant advanced melanoma, with recent results demonstrating 5-year OS rates of 34% for DAB+TRAM^7^ and 31% for VEM+COBI.^8^ Whether disease control can be maintained indefinitely, especially without continued treatment, remains to be determined.

Given that a substantial proportion of patients with metastatic melanoma have *BRAF*-mutant disease, there is tremendous value in exploring the relative efficacy of therapeutic options for this patient population. However, results from prospective randomized clinical trials (RCTs) comparing immunotherapy and targeted therapy have not yet been reported. Therefore, clinicians must rely on indirect comparisons to assess long-term outcomes with these treatments. Although indirect comparison of therapies across RCTs can be inherently biased because of differences in patient populations and study designs, statistical methods can reduce this bias and increase the precision of the results. A widely accepted statistical approach for estimating the relative efficacy of treatments from different trials is the matching-adjusted indirect comparison (MAIC).^9^^,^^10^ In this approach, individual patient-level data (IPD) for a treatment of interest (index therapy) are compared with aggregate-level (summary) data for comparator therapies by adjusting for differences in patient populations between similarly designed trials. The MAIC methodology has been applied extensively in oncology to assess the effects of treatments for various tumor types.^11^, ^12^, ^13^, ^14^, ^15^, ^16^, ^17^, ^18^

The objective of this analysis was to evaluate the relative efficacy and safety of NIVO+IPI (index therapy) versus DAB+TRAM, ENCO+BINI, and VEM+COBI (comparators) in patients with *BRAF*-mutant advanced melanoma using MAICs. This analysis expands on a previously reported MAIC analysis that compared NIVO+IPI with DAB+TRAM and VEM+COBI in this patient population.^19^

## Patients and methods

### Evidence base

A systematic literature review (SLR) of published articles and medical congress abstracts was conducted to identify RCTs that enrolled patients with *BRAF*-mutant advanced melanoma treated with DAB+TRAM, ENCO+BINI, or VEM+COBI. Studies involving patients who were exposed to prior systemic therapy were not excluded. Study selection methods are described in the Appendix, and study selection criteria are listed in Supplementary Table S1, available at https://doi.org/10.1016/j.esmoop.2021.100050.

### MAIC

A MAIC analysis was used to compare OS and progression-free survival (PFS) between NIVO+IPI and DAB+TRAM, ENCO+BINI, and VEM+COBI (Supplementary Figure S1, available at https://doi.org/10.1016/j.esmoop.2021.100050). IPD for NIVO+IPI were derived from the *BRAF*-mutant cohort in the 5-year follow-up of CheckMate 067^6^ and were restricted to match the inclusion/exclusion criteria of the comparator trials. Baseline characteristics included in the MAIC were age (>55, >56, or >57 years, based on medians reported in the comparator trials), sex, Eastern Cooperative Oncology Group (ECOG) performance status (PS; 0 or ≥1), lactate dehydrogenase (LDH) level [above or below the upper limit of normal (ULN)], and metastatic stage (M1b or M1c using the American Joint Committee on Cancer staging system seventh edition^2^). IPD for NIVO+IPI were weighted by adjusting for covariates known to impact treatment outcomes (effect modifiers). The impact of weighting was measured using Kish's effective sample size (ESS).^20^

Treatment effects were estimated for the overall study period using a Cox proportional hazards model, which determined hazard ratios (HRs) and corresponding confidence intervals (CIs) for the OS and PFS Kaplan–Meier curves. In the event that the proportional hazards assumption was violated, treatment effects were estimated for specific monthly time points and 12-month averages using a time-varying model, which determined HRs and corresponding 95% credible intervals (CrIs) using fractional polynomials. Models of varying complexity were tested and ranked according to deviance information criteria. Curve fit and extrapolation beyond the trial data were subsequently evaluated, and less complex models were used if they had a goodness of fit comparable to the more complex models. Relative safety was assessed by comparing the incidence of grade 3 or 4 treatment-related adverse events (TRAEs) between NIVO+IPI and the comparator therapies. The safety analysis was restricted to the weighted population with *BRAF*-mutant melanoma, and statistical significance was determined by calculating odds ratios (ORs) and corresponding 95% CrIs. All analyses were conducted using JAGS (v4.3.0) and programmed in R (v3.6.1).

## Results

### Evidence base

The analysis set for the MAIC comprised IPD from CheckMate 067^6^ for NIVO+IPI and data from seven publications reporting the results of three comparator RCTs: COMBI-d/v (NCT01584648 and NCT01597908, respectively) for DAB+TRAM,^7^^,^^21^ COLUMBUS (NCT01909453) for ENCO+BINI,^22^, ^23^, ^24^ and coBRIM (NCT01689519) for VEM+COBI^8^^,^^25^ (Supplementary Table S2 and Figure S2, available at https://doi.org/10.1016/j.esmoop.2021.100050). CheckMate 067 and the comparator trials had similar study designs, and OS and PFS results were available from all the comparator trials. Grade 3 or 4 TRAEs were not evaluated in the COLUMBUS trial^22^, ^23^, ^24^ and therefore were not available for ENCO+BINI. Although safety outcomes were evaluated in the COMBI-v trial, only all-cause and select adverse event (AE) rates were reported^8^; therefore, safety results (grade 3 or 4 TRAEs) for DAB+TRAM were obtained only from the COMBI-d trial.^21^

Baseline characteristics of patients treated with NIVO+IPI or the BRAF/MEK inhibitors in the RCTs are shown in Table 1. CheckMate 067 enrolled patients regardless of *BRAF*-mutation status.^6^ Of the 314 patients treated with NIVO+IPI in CheckMate 067, 103 had known *BRAF*-mutant disease and were included in this analysis. The DAB+TRAM, ENCO+BINI, and VEM+COBI groups from the comparator trials included 563, 192, and 247 patients, respectively. After weighting the data, ESSs for the comparisons of NIVO+IPI with DAB+TRAM, ENCO+BINI, and VEM+COBI were 91.68, 93.81, and 91.69, respectively. Baseline characteristics of the subgroup of patients with *BRAF*-mutant disease who received NIVO+IPI in CheckMate 067 were similar to those of patients receiving DAB+TRAM, ENCO+BINI, or VEM+COBI in the comparator trials. However, compared with the comparator trials, CheckMate 067 had larger percentages of patients aged over 55 years and with an ECOG PS of 0, as well as a smaller percentage with stage M1b or M1c disease.

**Table 1:** Baseline characteristics of patients treated with nivolumab plus ipilimumab or the BRAF/MEK inhibitors in randomized controlled trials with advanced melanoma

	NIVO+IPI (CheckMate 067)^6^ ITT population (*n* = 314)	NIVO+IPI (CheckMate 067),^6^*BRAF*-mutant patients (*n* = 103)	DAB+TRAM (COMBI-d/v)^8^^,^^21^ (*n* = 56)	ENCO+BINI (COLUMBUS)^22^, ^23^, ^24^ (*n* = 192)	VEM+COBI (coBRIM)^9^^,^^25^ (*n* = 247)
ESS for CheckMate 067	–	–	91.68	93.81	91.69
Age, %					
>55 years	63.7	56.3	50.0	–	–
>56 years	62.1	54.4	–	–	50.0
>57 years	58.6	48.5	–	50.0	–
Male, %	65.6	62.1	56.8	60.0	59.0
ECOG PS ≥1, %	26.4	22.3	27.9	29.0	24.0
M stage, %					
M1b	22.6	13.6	18.5	18.0	16.0
M1c	58.9	58.3	64.5	64.0	59.0
LDH > ULN, %	36.3	32.0	34.7	29.0	46.0

DAB+TRAM, dabrafenib plus trametinib; ECOG PS, Eastern Cooperative Oncology Group performance status; ENCO+BINI, encorafenib plus binimetinib; ESS, effective sample size; ITT, intention-to-treat; LDH, lactate dehydrogenase; M stage, metastasis stage; NIVO+IPI, nivolumab plus ipilimumab; ULN, upper limit of normal; VEM+COBI, vemurafenib plus cobimetinib.

**Figure 1:**
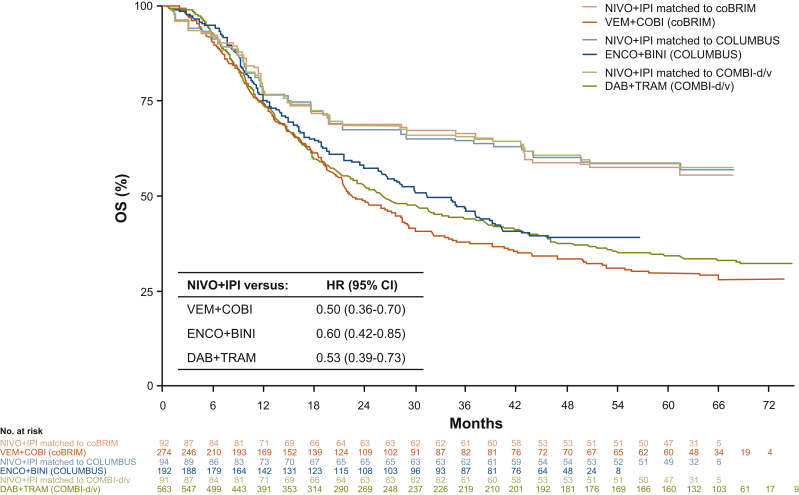
Matching-adjusted indirect comparison of overall survival with nivolumab plus ipilimumab versus BRAF/MEK inhibitors. Hazard ratios and confidence intervals were determined using a Cox proportional hazards model analysis. CI, confidence interval; DAB+TRAM, dabrafenib plus trametinib; ENCO+BINI, encorafenib plus binimetinib; HR, hazard ratio; NIVO+IPI, nivolumab plus ipilimumab; OS, overall survival; VEM+COBI, vemurafenib plus cobimetinib.

**Figure 2:**
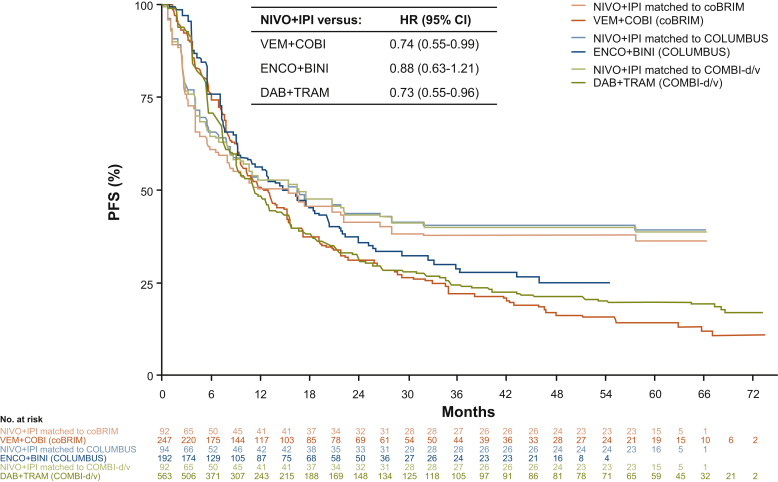
Matching-adjusted indirect comparison of progression-free survival with nivolumab plus ipilimumab versus BRAF/MEK inhibitors. Hazard ratios and confidence intervals were determined using a Cox proportional hazards model analysis. CI, confidence interval; DAB+TRAM, dabrafenib plus trametinib; ENCO+BINI, encorafenib plus binimetinib; HR, hazard ratio; NIVO+IPI, nivolumab plus ipilimumab; PFS, progression-free survival; VEM+COBI, vemurafenib plus cobimetinib.

Observed (unmatched) OS and PFS Kaplan–Meier curves for NIVO+IPI in the *BRAF*-mutant melanoma cohort in CheckMate 067 and those weighted (matched) to the comparator trials are shown in Supplementary Figures S3 and S4, respectively, available at https://doi.org/10.1016/j.esmoop.2021.100050. The matched OS curves fell slightly below the observed OS curve (Supplementary Figure S3, available at https://doi.org/10.1016/j.esmoop.2021.100050). The matched NIVO+IPI OS and PFS curves were used in the MAICs (Figures 1 and 2).

### Analysis of OS

Cox proportional hazards model analysis of OS demonstrated statistically significant reductions in the risk of death of patients treated with NIVO+IPI compared with those treated with DAB+TRAM (HR = 0.53; 95% CI, 0.39-0.73), ENCO+BINI (HR = 0.60; 95% CI, 0.42-0.85), or VEM+COBI (HR = 0.50; 95% CI, 0.36-0.70) (Table 2). Kaplan–Meier OS curves for NIVO+IPI (weighted) and for each of the three comparators are shown in Figure 1. However, the proportional hazards assumption was violated in all of the comparisons in the OS analysis, and a time-varying analysis was therefore performed.

**Table 2:** Matching-adjusted indirect comparison of overall survival with nivolumab plus ipilimumab versus BRAF/MEK inhibitors: Cox proportional hazards and time-varying model hazard ratios^a^

Comparator	HR (95% CI), overall study period	HR (95% CrI), 0-12 months	HR (95% CrI), 13-60 months
DAB+TRAM	0.53 (0.39-0.73)	0.93 (0.54-1.49)	0.33 (0.19-0.54)
ENCO+BINI	0.60 (0.42-0.85)	0.93 (0.53-1.58)	0.40 (0.23-0.68)
VEM+COBI	0.50 (0.36-0.70)	0.78 (0.45-1.31)	0.41 (0.27-0.60)

CI, confidence interval; CrI, credible interval; DAB+TRAM, dabrafenib plus trametinib; ENCO+BINI, encorafenib plus binimetinib; HR, hazard ratio; VEM+COBI, vemurafenib plus cobimetinib.

a Estimates for the overall time period were from the Cox proportional hazards model, and estimates for the time periods of 0-12 months and 13-60 months were from the time-varying HR model; model selections based on a deviance information criterion were scale and first shape (p0 = 0, p1 = 0) versus DAB+TRAM, scale and second shape (p0 = 1, p1 = 0) versus ENCO+BINI, and scale and second shape (p0 = 1, p1 = −1) versus VEM+COBI.

In the time-varying analysis, the risk for death was similar between NIVO+IPI and BRAF/MEK inhibitor treatment from 0 to 12 months (Table 2). However, NIVO+IPI treatment was associated with a significant reduction in the risk of death after 12 months compared with all three comparators. Similar trends were observed with a time-varying model that estimated HRs at discreet 3-month or 6-month landmarks, in which HRs favored NIVO+IPI over all three comparators beginning at 12 months and at every time point thereafter (Supplementary Table S3, available at https://doi.org/10.1016/j.esmoop.2021.100050). In each of the three comparisons, HRs decreased over time, suggesting ongoing improvement in OS benefit with NIVO+IPI. Because only 48-month follow-up data were available from the COLUMBUS trial^22^, ^23^, ^24^ at the time of this analysis, OS outcomes for ENCO+BINI were extrapolated to 60 months.

### Analysis of PFS

Cox proportional hazards model analysis of PFS demonstrated statistically significant reductions in the risk of progression or death for patients treated with NIVO+IPI compared with those treated with DAB+TRAM (HR = 0.73; 95% CI, 0.55-0.96) and VEM+COBI (HR = 0.74; 95% CI, 0.55-0.99) (Table 3). However, the difference in the risk of progression or death between NIVO+IPI and ENCO+BINI was not statistically significant (HR = 0.88; 95% CI, 0.63-1.21). Kaplan–Meier PFS curves for NIVO+IPI (weighted) and for each of the three comparators are shown in Figure 2. A time-varying analysis was performed because the proportional hazards assumption was violated for all three comparisons in the PFS analysis.

**Table 3:** Matching-adjusted indirect comparison of progression-free survival with nivolumab plus ipilimumab versus BRAF/MEK inhibitors: Cox proportional hazards and time-varying model hazard ratios^a^

Comparator	HR (95% CI), overall study period	HR (95% CrI), 0-12 months	HR (95% CrI), 13-60 months
DAB+TRAM	0.73 (0.55-0.96)	0.93 (0.68-1.24)	0.22 (0.12-0.41)
ENCO+BINI	0.88 (0.63-1.21)	1.19 (0.83-1.71)	0.21 (0.10-0.43)
VEM+COBI	0.74 (0.55-0.99)	1.06 (0.76-1.47)	0.17 (0.09-0.32)

CI, confidence interval; CrI, credible interval; DAB+TRAM, dabrafenib plus trametinib; ENCO+BINI, encorafenib plus binimetinib; HR, hazard ratio; VEM+COBI, vemurafenib plus cobimetinib.

a Estimates for the overall time period were from the Cox proportional hazards model, and estimates for the time periods of 0-12 months and 13-60 months were from the time-varying HR model; model selections based on a deviance information criterion were scale and first shape (p0 = 0, p1 = 0) versus DAB+TRAM, scale and second shape (p0 = 1, p1 = 0) versus ENCO+BINI, and scale and second shape (p0 = 1, p1 = −1) versus VEM+COBI.

The time-varying analysis showed no significant difference in the reduction in risk of progression or death based on HRs averaged over the time period between 0 and 12 months (Table 3). However, NIVO+IPI was associated with significant reductions in the risk of progression or death after 12 months compared with all three comparators. The time-varying analysis estimating monthly HRs showed similar trends, with a significant difference favoring NIVO+IPI over each of the three comparators beginning at 12 months and at every time point thereafter (Supplementary Table S4, available at https://doi.org/10.1016/j.esmoop.2021.100050). As in the OS analysis, PFS outcomes for ENCO+BINI were extrapolated to 60 months.

### Analysis of safety

Among patients with *BRAF*-mutant melanoma treated with NIVO+IPI in CheckMate 067, 66% (68/103) experienced a grade 3 or 4 TRAE. After weighting the data, ESSs were 83.3 and 91.7 for the safety comparisons between NIVO+IPI and DAB+TRAM (from the COMBI-d trial)^21^ or VEM+COBI (from the coBRIM trial),^8^ respectively. The weighted incidence of grade 3 or 4 TRAEs was significantly higher with NIVO+IPI than with DAB+TRAM [65% (54.0/83.3) versus 48% (101/211); OR = 2.01; 95% CrI, 1.19-3.39] but similar between NIVO+IPI and VEM+COBI [66% (60.9/91.7) versus 76% (188/248); OR = 0.63; 95% CrI, 0.38-1.07]. The safety comparison between NIVO+IPI and ENCO+BINI could not be carried out because grade 3 or 4 TRAEs were not evaluated in the COLUMBUS trial.^22^, ^23^, ^24^

## Discussion

The introduction of immunotherapy and targeted therapy has transformed the treatment of patients with *BRAF*-mutant advanced melanoma, but there is a lack of comparative data from RCTs, making treatment decisions difficult. Consequently, a MAIC analysis was conducted to estimate the relative efficacy and safety of the immunotherapy combination of NIVO+IPI compared with the targeted therapy combinations of DAB+TRAM, ENCO+BINI, and VEM+COBI. This MAIC used the longest follow-up data available for NIVO+IPI treatment (5-year minimum follow-up results from CheckMate 067^6^) and the most current data available from the comparator trials at the time of the analysis.^7^^,^^8^^,^^21^, ^22^, ^23^, ^24^, ^25^

This MAIC analysis demonstrated OS and PFS benefits with NIVO+IPI compared with BRAF/MEK inhibitors among patients with *BRAF*-mutant advanced melanoma. NIVO+IPI was associated with significant OS benefits compared with DAB+TRAM, ENCO+BINI, and VEM+COBI, and significant PFS benefits compared with DAB+TRAM and VEM+COBI, while the overall treatment effect for PFS in comparison with ENCO+BINI was not statistically significant. OS and PFS benefits were durable, with the magnitude of the treatment effects increasing over time. In the first 12 months after treatment initiation, outcomes for NIVO+IPI and the BRAF/MEK inhibitors were similar. However, beginning at 12 months, statistically significant improvement in OS and PFS were evident with NIVO+IPI compared with all of the BRAF/MEK inhibitor combinations. In particular, PFS curves for NIVO+IPI were essentially flat after 18 months, suggesting durability of response, while continuing to decrease for the BRAF/MEK inhibitors.

In the safety analysis involving grade 3 or 4 TRAEs, DAB+TRAM appeared to be better tolerated than NIVO+IPI, whereas NIVO+IPI and VEM+COBI demonstrated comparable safety outcomes. Using TRAEs to assess comparative safety, however, may be challenging given that commonly reported TRAEs can vary across therapeutic classes with regard to AE type, onset, duration, and response to management strategies. For example, BRAF/MEK inhibitors are usually associated with moderate TRAEs that resolve or diminish rapidly after dose reduction or treatment interruption.^26^ In contrast, immunotherapies have a propensity to elicit immune-mediated adverse events, which may have a prolonged duration in some instances and often require treatment with corticosteroids.^27^, ^28^, ^29^ It is also important to note that different adverse event categories may differentially impact quality of life, impeding safety comparisons across therapeutic classes. Therefore, the overall safety profile of a treatment, along with efficacy benefits, should be considered when selecting therapeutic options for patients with *BRAF*-mutant advanced melanoma.

This analysis expands on a previously reported MAIC analysis comparing NIVO+IPI with targeted therapy, specifically DAB+TRAM and VEM+COBI, in patients with *BRAF*-mutant advanced melanoma.^19^ In that earlier analysis, data for NIVO+IPI were pooled from CheckMate 067 (4-year follow-up) and the phase II CheckMate 069 study, and data for DAB+TRAM and VEM+COBI were derived from the COMBI-d/v and coBRIM trials, respectively. Results from that analysis were similar to the results presented here, with NIVO+IPI being associated with significantly improved OS compared with DAB+TRAM (HR = 0.64; 95% CI, 0.46-0.89) or VEM+COBI (HR = 0.56; 95% CI, 0.36-0.89) in Cox model analyses. As in the current study, OS outcomes were similar between NIVO+IPI and the comparators early in treatment (0-12 months), with benefits associated with NIVO+IPI emerging after 12 months of treatment. Similar results were noted with PFS. As in the current study, grade 3 or 4 TRAEs were reported significantly more often with NIVO+IPI than with DAB+TRAM (54% versus 32%; OR = 2.6; 95% CI, 1.8-3.6) but similarly with NIVO+IPI and VEM+COBI (54% versus 59%; OR = 0.8; 95% CI, 0.6-1.1). Findings from the current MAIC analysis augment the results of the prior analysis by involving extended follow-up times, a newer BRAF/MEK inhibitor combination (ENCO+BINI), NIVO+IPI data only from CheckMate 067 (which reported results for a minimum follow-up of 5 years),^6^ and restriction of the safety analysis to patients with *BRAF*-mutant disease (the previous analysis did not involve restriction, only weighting).

### Strengths and limitations

The strengths of this analysis rest primarily in the application of IPD from a large, robust, RCT (CheckMate 067), with comparator evidence identified through an extensive SLR process. This analysis was based on good practices in the field of indirect comparison research, with application of extensive restrictions and weighting of IPD to establish a comparable patient cohort.^9^

As with any indirect comparison approach, the MAIC analysis reported here was associated with limitations. Despite population restrictions and weighting, this analysis accounted only for known, measured, and reported effect modifiers, and unobserved differences between the trials may have confounded the results. However, the similarity of the study designs provided some confidence that the observed outcomes were not simply the result of differences between studies. In addition, the statistical power of MAIC analyses can be diminished if the ESS becomes small when too few patients are assigned a high weight. Nonetheless, the impact of weighting by ESS in this analysis suggested that the comparisons were sufficiently powered. This analysis may also have been limited by the lack of consideration of other efficacy outcomes, such as duration of response and survival among patients reaching complete and/or partial tumor response. The current analysis also did not evaluate comparisons by patient subgroups of interest (e.g. those with elevated baseline LDH levels) due to limited patient numbers, which precluded a robust subgroup analysis. Moreover, results of the comparison of NIVO+IPI and ENCO+BINI should be interpreted with caution as only 4-year follow-up data were available from the COLUMBUS trial at the time of this analysis, necessitating extrapolation of OS and PFS outcomes to 5 years. However, because the 4-year OS and PFS data from COLUMBUS were mature, results of the comparison are unlikely to change with the use of longer follow-up data. Furthermore, the weighted NIVO+IPI OS curves in all three comparisons fell slightly below the observed OS curve, suggesting that patients enrolled in the targeted therapy trials may have had a worse prognosis than those enrolled in CheckMate 067. This finding underscores the bias involved in making naive (unadjusted) comparisons across trials. It is also unclear how the results of this MAIC should be interpreted relative to outcomes with the combination of atezolizumab (a programmed deathligand 1 inhibitor) and VEM+COBI, which was recently approved as a first-line treatment of *BRAF*-mutant advanced melanoma.^30^ Data for the combination of atezolizumab and VEM+COBI were not available at the time of this analysis and did not appear to be mature; future MAICs should include this new therapeutic option.

An important limitation of indirect comparisons of cancer treatments is the inability to control for differences in the use of subsequent therapy between trials, which may influence OS outcomes unequally. Selection of subsequent therapy in oncology trials may be based on several factors, including the availability of specific treatment options at the time of the study and at the geographic location of the study site. Geographic differences existed between the trials included in this analysis. Patients enrolled in Europe comprised 74% and 81% of the coBRIM^25^ and COLUMBUS^22^^,^^23^ trial populations, respectively, compared with 56% of the NIVO+IPI treatment arm in CheckMate 067 (geographic distribution information was not available for COMBI-v/d).^29^ Perhaps because of these geographic differences, subsequent therapy differed between the comparator trials and CheckMate 067. In the COMBI-v/d,^7^ COLUMBUS,^31^ and coBRIM^32^ trials, immunotherapy was the most prevalent subsequent therapy. Although the start of enrollment in the COMBI-v/d trials preceded that of the coBRIM and COLUMBUS trials by approximately 1 year, use of subsequent treatment was similar in these trials. In contrast, the most frequently used subsequent therapy among patients with *BRAF*-mutant melanoma in CheckMate 067 was BRAF inhibitors (data on file). Therefore, this MAIC analysis should not be considered a true comparison of treatment sequencing but rather a comparison of first-line treatment choices in which OS may have been affected by the use of subsequent therapy. However, the inability to control for differences in the use of subsequent therapy may have been offset by the use of PFS outcomes in this analysis. PFS is considered to be less affected by subsequent therapy than OS, and the PFS results in this analysis demonstrated statistically significant differences favoring NIVO+IPI over the comparators after 12 months.

## Conclusion

In the absence of head-to-head evidence from RCTs, this MAIC provides insights into the comparative efficacy and safety of therapies approved by the US FDA for patients with *BRAF*-mutant advanced melanoma. This analysis demonstrated durable OS and PFS benefits among patients with *BRAF*-mutant melanoma treated with NIVO+IPI compared with those treated with DAB+TRAM, ENCO+BINI, or VEM+COBI. These benefits increased over time, with significant treatment effects with NIVO+IPI emerging after 12 months, suggesting that NIVO+IPI had a more durable benefit than the targeted therapies. These findings supplement the long-term efficacy and safety results reported in RCTs and may provide clinicians with additional information relevant to the selection of treatments for *BRAF*-mutant advanced melanoma. Results of the ongoing phase III DREAMseq trial (NCT02224781) and the phase II SECOMBIT trial (NCT02631447), which are comparing sequential regimens of NIVO+IPI plus DAB+TRAM or ENCO+BINI, respectively, may confirm the optimal sequence of initial therapy for patients with *BRAF*-mutant advanced melanoma.

## References

[1] Garbe C., Eigentler T.K., Keilholz U. (2011). Systematic review of medical treatment in melanoma: current status and future prospects. Oncologist.

[2] Balch C.M., Gershenwald J.E., Soong S.J. (2009). Final version of 2009 AJCC melanoma staging and classification. J Clin Oncol.

[3] Schummer P., Schilling B., Gesierich A. (2020). Long-term outcomes in *BRAF*-mutated melanoma treated with combined targeted therapy or immune checkpoint blockade: are we approaching a true cure?. Am J Clin Dermatol.

[4] Moreira A., Heinzerling L., Bhardwaj N., Friedlander P. (2021). Current melanoma treatments: where do we stand?. Cancers (Basel).

[5] Larkin J., Chiarion-Sileni V., Gonzalez R. (2015). Combined nivolumab and ipilimumab or monotherapy in untreated melanoma. N Engl J Med.

[6] Larkin J., Chiarion-Sileni V., Gonzalez R. (2019). Five-year survival with combined nivolumab and ipilimumab in advanced melanoma. N Engl J Med.

[7] Robert C., Grob J.J., Stroyakovskiy D. (2019). Five-year outcomes with dabrafenib plus trametinib in metastatic melanoma. N Engl J Med.

[8] McArthur G, Dreno B, Larkin J. 5-year survival update of cobimetinib plus vemurafenib in *BRAFV600* mutation-positive advanced melanoma: final analysis of the coBRIM study. Paper presented at the 16th International Congress of the Society for Melanoma Research; November 20-23, 2019; Salt Lake City, UT.

[9] Phillippo D.M., Ades A.E., Dias S. (2018). Methods for population-adjusted indirect comparisons in health technology appraisal. Med Decis Making.

[10] Signorovitch J.E., Sikirica V., Erder M.H. (2012). Matching-adjusted indirect comparisons: a new tool for timely comparative effectiveness research. Value Health.

[11] Chowdhury S., Oudard S., Uemura H. (2020). Matching-adjusted indirect comparison of the efficacy of apalutamide and enzalutamide with ADT in the treatment of non-metastatic castration-resistant prostate cancer. Adv Ther.

[12] Levy M.Y., McGarry L.J., Huang H. (2018). Benefits and risks of ponatinib versus bosutinib following treatment failure of two prior tyrosine kinase inhibitors in patients with chronic phase chronic myeloid leukemia: a matching-adjusted indirect comparison. Curr Med Res Opin.

[13] Li J., Knoll S., Bocharova I. (2018). Comparative efficacy of first-line ceritinib and crizotinib in advanced or metastatic anaplastic lymphoma kinase-positive non-small cell lung cancer: an adjusted indirect comparison with external controls. Curr Med Res Opin.

[14] Proskorovsky I., Benedict A., Negrier S. (2018). Axitinib, cabozanitib, or everolimus in the treatment of prior sunitinib-treated patients with metastatic renal cell carcinoma: results of matching-adjusted indirect comparison analyses. BMC Cancer.

[15] Song J., Ma Q., Gao W. (2019). Matching-adjusted indirect comparison of blinatumomab vs. inotuzumab ozogamicin for adults with relapsed/refractory acute lymphoblastic leukemia. Adv Ther.

[16] Tremblay G., Chandiwana D., Dolph M. (2018). Matching-adjusted indirect treatment comparison of ribociclib and palbociclib in HR+, HER2− advanced breast cancer. Cancer Manag Res.

[17] Van Sanden S., Baculea S., Diels J., Cote S. (2017). Comparative efficacy of ibrutinib versus obinutuzumab + chlorambucil in first-line treatment of chronic lymphocytic leukemia: a matching-adjusted indirect comparison. Adv Ther.

[18] Van Sanden S., Ito T., Diels J. (2018). Comparative efficacy of daratumumab monotherapy and pomlidomide plus low-dose dexamethasone in the treatment of multiple myeloma: a matching-adjusted indirect comparison. Oncologist.

[19] Atkins M.B., Tarhini A., Rael M. (2019). Comparative efficacy of combination immunotherapy and targeted therapy in the treatment of *BRAF*-mutant advanced melanoma: a matching-adjusted indirect comparison. Immunotherapy.

[20] Kish L. (1965). Survey Sampling.

[21] Flaherty K., Davies M.A., Grob J.J. (2016). Genomic analysis and 3-y efficacy and safety update of COMBI-d: A phase 3 study of dabrafenib (D) +trametinib (T) vs D monotherapy in patients (pts) with unresectable or metastatic *BRAF* V600E/K-mutant cutaneous melanoma. J Clin Oncol.

[22] Dummer R., Ascierto P.A., Gogas H.J. (2018). Encorafenib plus binimetinib versus vemurafenib or encorafenib in patients with *BRAF*-mutant melanoma (COLUMBUS): a multicentre, open-label, randomised phase 3 trial. Lancet Oncol.

[23] Dummer R., Ascierto P.A., Gogas H.J. (2018). Overall survival in patients with BRAF-mutant melanoma receiving encorafenib plus binimetinib versus vemurafenib or encorafenib (COLUMBUS): a multicentre, open-label, randomised, phase 3 trial. Lancet Oncol.

[24] Liszkay G., Gogas H., Mandala M. (2019). Update on overall survival in COLUMBUS: a randomized phase III trial of encorafenib (ENCO) plus binimetinib (BINI) versus vemurafenib (VEM) or ENCO in patients with *BRAF* V600-mutant melanoma. J Clin Oncol.

[25] Larkin J., Ascierto P.A., Dréno B. (2014). Combined vemurafenib and cobimetinib in *BRAF*-mutated melanoma. N Engl J Med.

[26] Heinzerling L., Eigentler T.K., Fluck M. (2019). Tolerability of BRAF/MEK inhibitor combinations: adverse event evaluation and management. ESMO Open.

[27] Parakh S., Randhawa M., Nguyen B. (2019). Real-world efficacy and toxicity of combined nivolumab and ipilimumab in patients with metastatic melanoma. Asia Pac J Clin Oncol.

[28] Shoushtari A.N., Friedman C.F., Navid-Azarbaijani P. (2018). Measuring toxic effects and time to treatment failure for nivolumab plus ipilimumab in melanoma. JAMA Oncol.

[29] Wolchok J.D., Chiarion-Sileni V., Gonzalez R. (2017). Overall survival with combined nivolumab and ipilimumab in advanced melanoma. N Engl J Med.

[30] Gutzmer R., Stroyakovskiy D., Gogas H. (2020). Atezolizumab, vemurafenib, and cobimetinib as first-line treatment for unresectable advanced BRAF^V600^ mutation-positive melanoma (IMspire150): primary analysis of the randomised, double-blind, placebo-controlled, phase 3 trial. Lancet.

[31] Ascierto P.A., Dummer R., Gogas H.J. (2020). Update on tolerability and overall survival in COLUMBUS: landmark analysis of a randomised phase 3 trial of encorafenib plus binimetinib vs vemurafenib or encorafenib in patients with BRAF V600-mutant melanoma. Eur J Cancer.

[32] Dréno B, Ascierto PA, McArthur GA, et al. Efficacy and safety of cobimetinib combined with vemurafenib in patients with *BRAF*^V600^ mutation-positive metastatic melanoma: analysis from the 4-year extended follow-up of the phase 3 coBRIM study. Paper presented at: 2018 ASCO (American Society of Clinical Oncology) Annual Meeting; June 1-5, 2018; Chicago, IL.

