# Supplementary material for: A matching-adjusted indirect comparison of combination nivolumab plus ipilimumab with BRAF plus MEK inhibitors for the treatment of BRAF-mutant advanced melanoma☆
Source: ESMO Open. 2021 Feb 6;6(2):100050. doi: 10.1016/j.esmoop.2021.100050 (PMC7872980; doi:10.1016/j.esmoop.2021.100050)

SUPPLEMENT

A matching-adjusted indirect comparison of combination nivolumab plus ipilimumab with BRAF plus MEK inhibitors for the treatment of *BRAF*-mutant advanced melanoma

Ahmad A. Tarhini,[1]* Kabirraaj Toor,[2] Keith Chan,[2] David F. McDermott,[3] Peter Mohr,[4] James Larkin,[5] F. Stephen Hodi,[6] Cho-Han Lee,[7] Jasmine I. Rizzo,[8] Helen Johnson,[9] Andriy Moshyk,[10] Sumati Rao,[7] Srividya Kotapati,[11] Michael B. Atkins[12]

[1]Department of Cutaneous Oncology, H. Lee Moffitt Cancer Center and Research Institute, Tampa, USA; [2]Evidence Synthesis and Decision Modeling, Precision HEOR, Vancouver, Canada; [3]Medical Oncology, Beth Israel Deaconess Medical Center and Harvard Medical School, Boston, USA; [4]Department of Dermatology, Elbe Kliniken Buxtehude, Buxtehude, Germany; [5]Medical Oncology, The Royal Marsden Hospital, London, UK; [6]Medical Oncology, Dana-Farber/Harvard Cancer Center, Boston, USA; [7]US Health Economics and Outcome Research, Metastatic Melanoma, Bristol Myers Squibb, Princeton, USA; [8]Oncology Clinical Development, Bristol Myers Squibb, Princeton, USA; [9]Worldwide Health Economics and Outcomes Research, Melanoma, Bristol Myers Squibb, Uxbridge, UK; [10]Worldwide Health Economics and Outcomes Research, Melanoma, Bristol Myers Squibb, Princeton, USA; [11]Worldwide Medical, Melanoma; Bristol Myers Squibb, Princeton, USA; [12]Medical Oncology, Georgetown Lombardi Comprehensive Cancer Center, Washington, DC, USA.

Appendix

A systematic literature review (SLR) was conducted in MEDLINE, EMBASE, and the Cochrane Register of Controlled Trials (CENTRAL) to identify randomized controlled trials of systemic therapies in adults with unresectable advanced (stage III/IV) melanoma that reported overall survival or grade 3 or 4 treatment-related adverse events. The search for published manuscripts was updated to January 2020. Searches of the medical literature databases were supplemented with hand searches of conference proceedings from the European Society of Medical Oncology (2017 to 2019), the American Society of Clinical Oncology (2017 to 2019), the Society for Melanoma Research Congress (2017 to 2019), and the Society for Immunotherapy of Cancer (2018 to 2019). Two reviewers, working independently and in duplicate, reviewed all abstracts and proceedings against the study design, population, and intervention selection criteria. The complete eligibility criteria (including outcomes) were then applied to the full-text publications of included abstracts. Data extraction of eligible studies was conducted independently and in duplicate by two reviewers. Discrepancies in the data extraction stages were resolved by discussion or the involvement of a third reviewer to provide arbitration, if necessary. The SLR process was conducted in accordance with PRISMA guidelines.[1] Because the current analysis focused on US Food and Drug Administration-approved treatments in which a connected network did not exist for the *BRAF*-mutant patient subgroup, comparators of interest for this particular matching-adjusted indirect comparison were DAB+TRAM, ENCO+BINI, and VEM+COBI. Individual patient data from the 5-year data cut of the CheckMate 067 trial (July 31, 2019) were provided from the study sponsor (Bristol Myers Squibb).

REFERENCE

1. Moher D, Liberati A, Tetzlaff J, *et al*. Preferred reporting items for systematic reviews and meta-analyses: the PRISMA statement. *PLoS Med* 2009;6:e1000097.

Supplementary Table S1 Study selection criteria[a]

Criteria	Description
Population	Adult patients with unresectable, advanced (stage III/IV) melanoma
Interventions	Any of the following treatments as monotherapy or in combination with other treatments:<br>• Nivolumab[b]<br>• Ipilimumab[b]<br>• Vemurafenib<br>• Dabrafenib<br>• Trametinib<br>• Cobimetinib<br>• Encorafenib<br>• Binimetinib
Comparators	Any of the following comparators:<br>• Placebo or best supportive care<br>• Any intervention of interest<br>• Any treatment that facilitates an indirect comparison
Outcomes	Studies must have reported report at least one of the following outcomes:<br>• OS<br>• PFS/time to progression<br>• Objective response rate
Study design	RCT
Language	English

[a]The following study characteristics, patient characteristics, intervention characteristics, and outcomes were extracted: treatment regimen, treatment dose, method of administration, frequency of administration, duration of treatment, and concomitant/background therapies. The following patient characteristics were extracted: sample size at baseline, age, gender, race and ethnicity, disease stage, performance status, genetic status, comorbidities, prior treatment experience, and lines of prior treatment. The following outcomes were extracted: response rate, OS, PFS, objective response rate, overall discontinuations, and discontinuations due to adverse events.

[b]The CheckMate 067 trial was the only data source for patients with *BRAF*-mutant melanoma treated with NIVO+IPI in the MAIC.

MAIC, matching-adjusted indirect comparison; NIVO+IPI, nivolumab plus ipilimumab; OS, overall survival; PFS, progression-free survival; RCT, randomized controlled trial.

Supplementary Table S2 Comparison of key design features of the CheckMate 067, COMBI-d/v, coBRIM, and COLUMBUS trials

	**CheckMate 067[1,2] (N=945)**	**COMBI-d[3,4] (N=423)**	**COMBI-v[4,5] (N=704)**	**coBRIM[6,7] (N=495)**	**COLUMBUS[8-10] (N=577)**
Treatments	**NIVO+IPI:** NIVO 1 mg/kg + IPI 3 mg/kg IV Q3W for 4 doses, then NIVO 3 mg/kg IV Q2W<br>**NIVO:** 3 mg/kg IV Q2W<br>**IPI:** 3 mg/kg IV Q3W for 4 doses	**DAB+TRAM:**<br>DAB 150 mg PO BID + TRAM 2 mg PO QD<br>**DAB:** 150 mg PO BID	**DAB+TRAM:**<br>DAB 150 mg PO BID + TRAM 2 mg PO QD<br>**VEM:** 960 mg PO BID	**VEM+COBI:**<br>VEM 960 mg PO BID + COBI 60 mg PO QD<br>**VEM:** 960 mg PO BID	**ENCO+BINI:**<br>ENCO 450 mg QD + BINI 45 mg BID<br>**ENCO:** 300 mg QD<br>**VEM:** 960 mg BID
Tumor assessment schedule	At 12 weeks, then every 6 weeks for 49 weeks, then every 12 weeks thereafter	Every 8 weeks to 56 weeks, then every 12 weeks thereafter		Every 8 weeks	Every 8 weeks for 24 months, then every 12 weeks thereafter
Median follow-up	54.6, 36.0, and 18.6 months for NIVO+IPI, NIVO, and IPI, respectively[2]	22 months for DAB+TRAM from COMBI-d and COMBI-v pooled[4]		21.2 months for VEM+COBI and 16.6 months for VEM[7]	48.6 months across all arms[10]
Melanoma diagnosis	Unresectable stage III or stage IV	Unresectable stage IIIC or stage IV			Locally advanced, unresectable or metastatic cutaneous melanoma or unknown primary melanoma stage IIIB, IIIC, or IV
BRAF status	Wild-type and mutant	Mutant			
Treatment history	No prior systemic treatment for advanced disease				Untreated patients or patients with progression on or after prior first-line immunotherapy for unresectable locally advanced or metastatic melanoma

BID, twice daily; BINI, binimetinib; COBI, cobimetinib; DAB, dabrafenib; ENCO, encorafenib; IPI, ipilimumab; IV, intravenous; NIVO, nivolumab; PO, by mouth; Q2W, every 2 weeks; Q3W, every 3 weeks; QD, once daily; TRAM, trametinib; VEM vemurafenib.

REFERENCES

1. Larkin J, Chiarion-Sileni V, Gonzalez R, *et al.* Combined nivolumab and ipilimumab or monotherapy in untreated melanoma. *N Engl J Med* 2015;373:23–34.
2. Larkin J, Chiarion-Sileni V, Gonzalez R, *et al.* Five-year survival with combined nivolumab and ipilimumab in advanced melanoma. *N Engl J Med* 2019;381:1535–46.
3. Flaherty K, Davies MA, Grob JJ, *et al*. Genomic analysis and 3-y efficacy and safety update of COMBI-d: a phase 3 study of dabrafenib (D) + trametinib (T) vs D monotherapy in patients (pts) with unresectable or metastatic *BRAF* V600E/K-mutant cutaneous melanoma. *J Clin Oncol* 2016;34(15 suppl):9502.
4. Robert C, Grob JJ, Stroyakovskiy D, *et al*. Five-year outcomes with dabrafenib plus trametinib in metastatic melanoma. *N Engl J Med* 2019;381:626–36.
5. Robert C, Karaszewska B, Schachter J, *et al.* Improved overall survival in melanoma with combined dabrafenib and trametinib. *N Engl J Med* 2015;372(1):30–39.
6. Larkin J, Ascierto PA, Dréno B, *et al*. Combined vemurafenib and cobimetinib in *BRAF*-mutated melanoma. *N Engl J Med* 2014;371:1867–76.
7. McArthur GA, Dréno B, Larkin J, *et al.* 5-year survival update of cobimetinib plus vemurafenib *BRAF* V600 mutation-positive advanced melanoma: final analysis of the coBRIM study. Presented at the 16th International Congress of the Society for Melanoma Research; November 20–23, 2019; Salt Lake City, USA.
8. Dummer R, Ascierto PA, Gogas HJ, *et al*. Encorafenib plus binimetinib versus vemurafenib or encorafenib in patients with BRAF-mutant melanoma (COLUMBUS): a multicentre, open-label, randomised phase 3 trial. *Lancet Oncol* 2018;19:603–15.
9. Dummer R, Ascierto PA, Gogas HJ, *et al.* Overall survival in patients with BRAF-mutant melanoma receiving encorafenib plus binimetinib versus vemurafenib or encorafenib (COLUMBUS): a multicentre, open-label, randomised, phase 3 trial. *Lancet Oncol* 2018;19:1315–27.
10. Liszkay G, Gogas H, Mandala M, *et al.* Update on overall survival in COLUMBUS: A randomized phase III trial of encorafenib (ENCO) plus binimetinib (BINI) versus vemurafenib (VEM) or ENCO in patients with *BRAF* V600–mutant melanoma. *J Clin Oncol* 2019;37(15 suppl):9512.

Supplementary Table S3 MAIC of OS with NIVO+IPI versus BRAF/MEK inhibitors: time-varying HRs[a]

NIVO+IPI versus:	HR (95% CrI)										
	**6 months**	**9 months**	**12 months**	**18 months**	**24 months**	**30 months**	**36 months**	**42 months**	**48 months**	**54 months**	**60 months**
DAB+TRAM	0.86 (0.53 to 1.34)	0.69 (0.47 to 0.98)	0.59 (0.41 to 0.81)	0.47 (0.32 to 0.66)	0.40 (0.26 to 0.60)	0.36 (0.21 to 0.56)	0.32 (0.18 to 0.54)	0.30 (0.16 to 0.52)	0.27 (0.14 to 0.50)	0.26 (0.13 to 0.49)	0.24 (0.11 to 0.48)
ENCO+BINI	0.88 (0.52 to 1.43)	0.73 (0.48 to 1.08)	0.64 (0.44 to 0.92)	0.54 (0.36 to 0.78)	0.47 (0.30 to 0.72)	0.43 (0.26 to 0.69)	0.39 (0.22 to 0.67)	0.37 (0.20 to 0.66)	0.34 (0.18 to 0.65)	0.33 (0.16 to 0.65)[b]	0.31 (0.15 to 0.64)[b]
VEM+COBI	0.60 (0.40 to 0.88)	0.52 (0.36 to 0.72)	0.48 (0.33 to 0.67)	0.44 (0.30 to 0.63)	0.42 (0.28 to 0.61)	0.41 (0.28 to 0.61)	0.41 (0.27 to 0.60)	0.40 (0.27 to 0.60)	0.40 (0.26 to 0.59)	0.40 (0.26 to 0.59)	0.40 (0.26 to 0.59)
**NIVO+IPI versus:**	1 to 12 months			**13 to 24 months**		**25 to 36 months**		**37 to 48 months**		**49 to 60 months**	
DAB+TRAM	0.93 (0.54 to 1.49)			0.47 (0.32 to 0.66)		0.35 (0.21 to 0.56)		0.29 (0.16 to 0.52)		0.26 (0.12 to 0.49)	
ENCO+BINI	0.93 (0.53 to 1.58)			0.53 (0.36 to 0.77)		0.42 (0.25 to 0.69)		0.36 (0.20 to 0.66)		0.33 (0.16 to 0.64)[b]	
VEM+COBI	0.78 (0.45 to 1.31)			0.44 (0.30 to 0.63)		0.41 (0.28 to 0.61)		0.40 (0.27 to 0.60)		0.40 (0.26 to 0.59)	

[a]Model selections based on deviance information criterion were scale and first shape (p0 = 0, p1 = 0) versus DAB+TRAM, scale and second shape (p0 = 1, p1 = 0) versus ENCO+BINI, and scale and second shape (p0 = 1, p1 = −1) versus VEM+COBI.
[b]Based on extrapolations beyond the trial data.
CrI, credible interval; DAB+TRAM, dabrafenib plus trametinib; ENCO+BINI, encorafenib plus binimetinib; HR, hazard ratio; MAIC, matching-adjusted indirect comparison; NIVO+IPI, nivolumab plus ipilimumab; OS, overall survival; VEM+COBI, vemurafenib plus cobimetinib.

Supplementary Table S4 MAIC of PFS with NIVO+IPI versus BRAF/MEK inhibitors: time-varying HRs[a]

NIVO+IPI versus:	HR (95% CrI)										
	**6 months**	**9 months**	**12 months**	**18 months**	**24 months**	**30 months**	**36 months**	**42 months**	**48 months**	**54 months**	**60 months**
DAB+TRAM	0.84 (0.62 to 1.12)	0.62 (0.44 to 0.84)	0.50 (0.33 to 0.70)	0.36 (0.22 to 0.56)	0.29 (0.16 to 0.48)	0.25 (0.13 to 0.43)	0.21 (0.11 to 0.39)	0.19 (0.09 to 0.37)	0.17 (0.08 to 0.34)	0.16 (0.07 to 0.33)	0.14 (0.06 to 0.31)
ENCO+BINI	1.06 (0.74 to 1.50)	0.73 (0.50 to 1.04)	0.56 (0.36 to 0.83)	0.38 (0.22 to 0.63)	0.29 (0.15 to 0.53)	0.24 (0.12 to 0.46)	0.20 (0.09 to 0.42)	0.17 (0.07 to 0.38)	0.15 (0.06 to 0.35)	0.14 (0.05 to 0.33)	0.13 (0.05 to 0.31)[b]
VEM+COBI	0.94 (0.67 to 1.28)	0.63 (0.44 to 0.87)	0.47 (0.31 to 0.68)	0.32 (0.19 to 0.50)	0.24 (0.13 to 0.41)	0.19 (0.10 to 0.35)	0.16 (0.08 to 0.31)	0.14 (0.06 to 0.28)	0.12 (0.05 to 0.25)	0.11 (0.05 to 0.23)	0.10 (0.04 to 0.22)

**NIVO+IPI versus:**	**1 to 12 months**	**13 to 24 months**	**25 to 36 months**	**37 to 48 months**	**49 to 60 months**
DAB+TRAM	0.93 (0.68 to 1.24)	0.36 (0.22 to 0.56)	0.24 (0.13 to 0.43)	0.19 (0.09 to 0.36)	0.16 (0.07 to 0.32)
ENCO+BINI	1.19 (0.83 to 1.71)	0.38 (0.22 to 0.63)	0.24 (0.11 to 0.46)	0.17 (0.07 to 0.38)	0.14 (0.05 to 0.33)[b]
VEM+COBI	1.06 (0.76 to 1.47)	0.32 (0.19 to 0.50)	0.19 (0.10 to 0.35)	0.14 (0.06 to 0.28)	0.11 (0.05 to 0.23)

[a]Model selections based on deviance information criterion were scale and first shape (p0 = 0, p1 = 0) versus DAB+TRAM, scale and second shape (p0 = 1, p1 = 0) versus ENCO+BINI, and scale and second shape (p0 = 1, p1 = −1) versus VEM+COBI.
[b]Base on extrapolations beyond the trial data.
CrI, credible interval; DAB+TRAM, dabrafenib plus trametinib; ENCO+BINI, encorafenib plus binimetinib; HR, hazard ratio; MAIC, matching-adjusted indirect comparison; NIVO+IPI, nivolumab plus ipilimumab; PFS, progression-free survival; VEM+COBI, vemurafenib plus cobimetinib.

Supplementary Figure S1 MAIC approach. ESS was used to quantify information lost during matching. DAB+TRAM, dabrafenib plus trametinib; ENCO+BINI, encorafenib plus binimetinib; ESS, effective sample size; HR, hazard ratio; MAIC, matching-adjusted indirect comparison; MT, mutant; NIVO+IPI, nivolumab plus ipilimumab; OR, odds ratio; OS, overall survival; PFS, progression-free survival; VEM+COBI, vemurafenib plus cobimetinib; WT, wild-type.

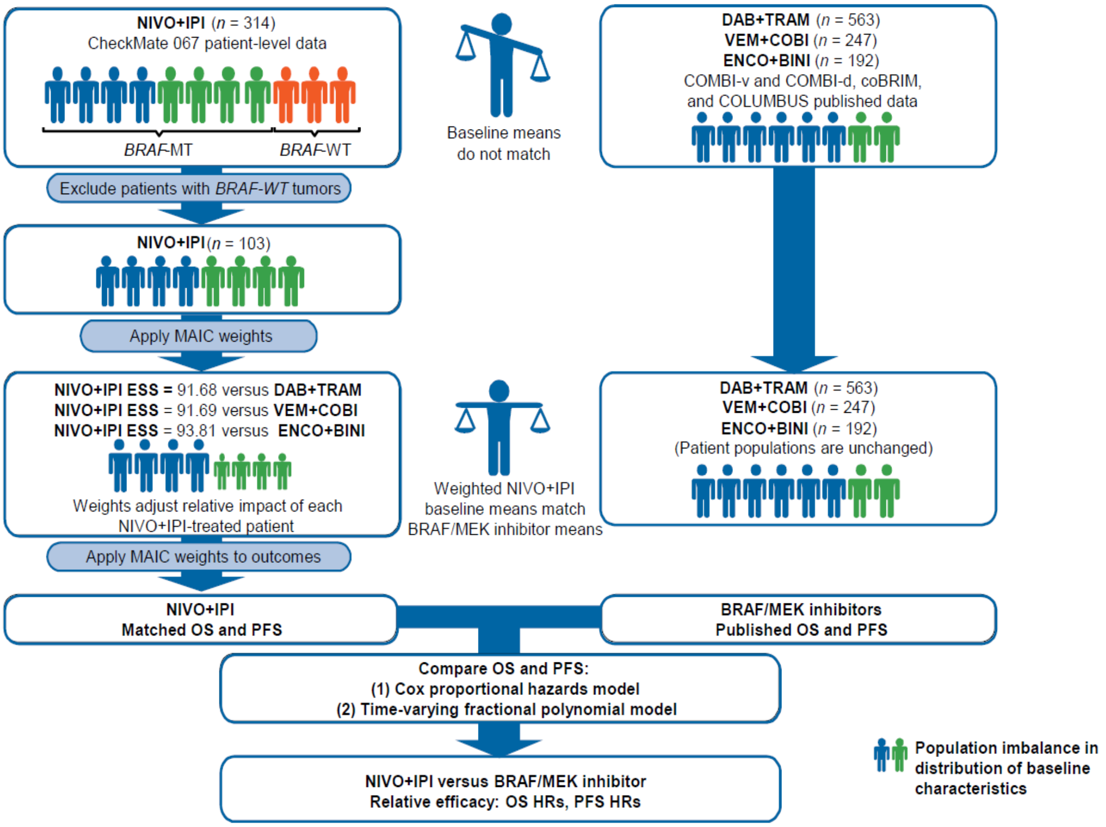

Supplementary Figure S2 PRISMA flow diagram. Studies excluded due to "study design" included economic models, observational studies, registry studies, and case studies (i.e., they were not RCTs); studies excluded as "other" included reviews, notes, editorials, and corrections (i.e., they were not full publications). IPD, individual patient-level data; RCT, randomized clinical trial; SLR, systematic literature review.

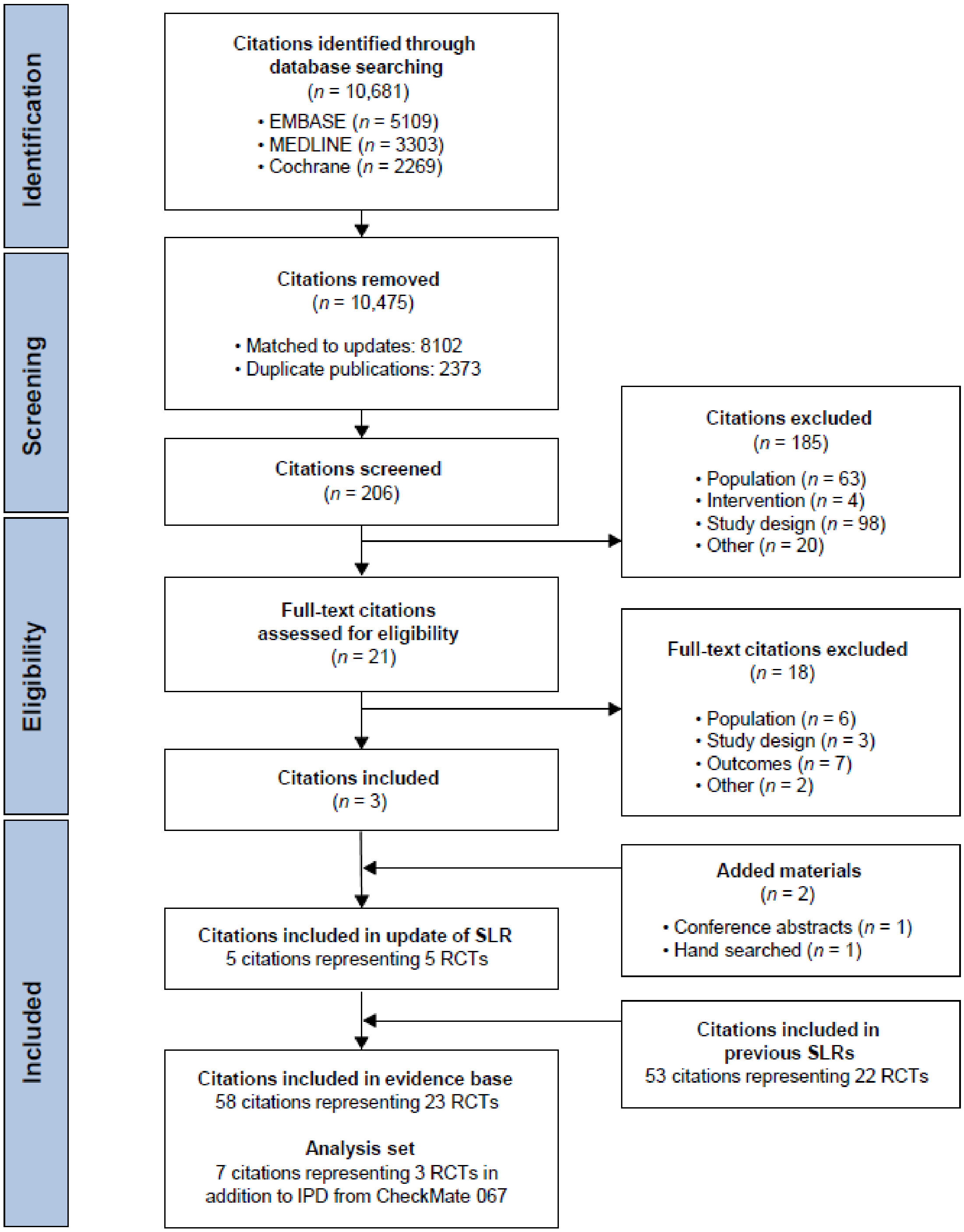

Supplementary Figure S3 OS with NIVO+IPI in CheckMate 067: observed versus MAIC-matched results. DAB+TRAM, dabrafenib plus trametinib; ENCO+BINI, encorafenib plus binimetinib; MAIC, matching-adjusted indirect comparison; NIVO+IPI, nivolumab plus ipilimumab; OS, overall survival; VEM+COBI, vemurafenib plus cobimetinib.

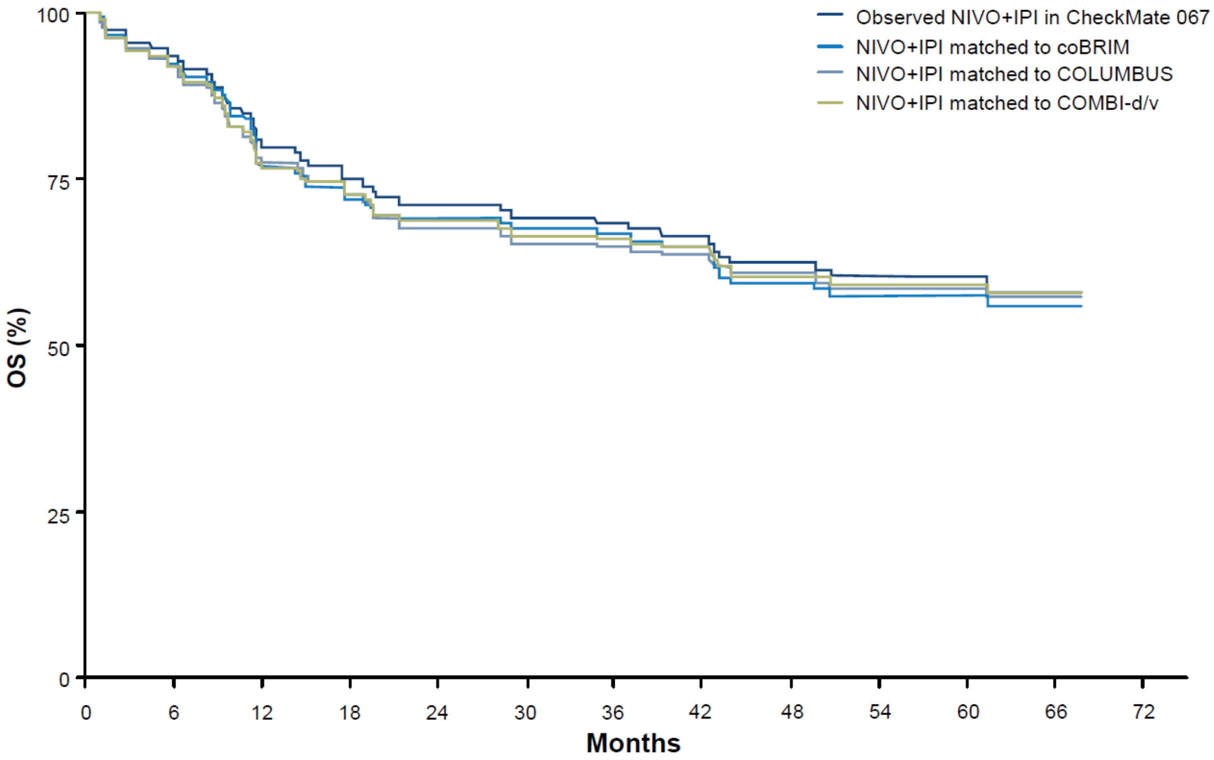

Supplementary Figure S4 PFS with NIVO+IPI in CheckMate 067: observed versus MAIC-matched results. DAB+TRAM, dabrafenib plus trametinib; ENCO+BINI, encorafenib plus binimetinib; MAIC, matching-adjusted indirect comparison; NIVO+IPI, nivolumab plus ipilimumab; PFS, progression-free survival; VEM+COBI, vemurafenib plus cobimetinib.

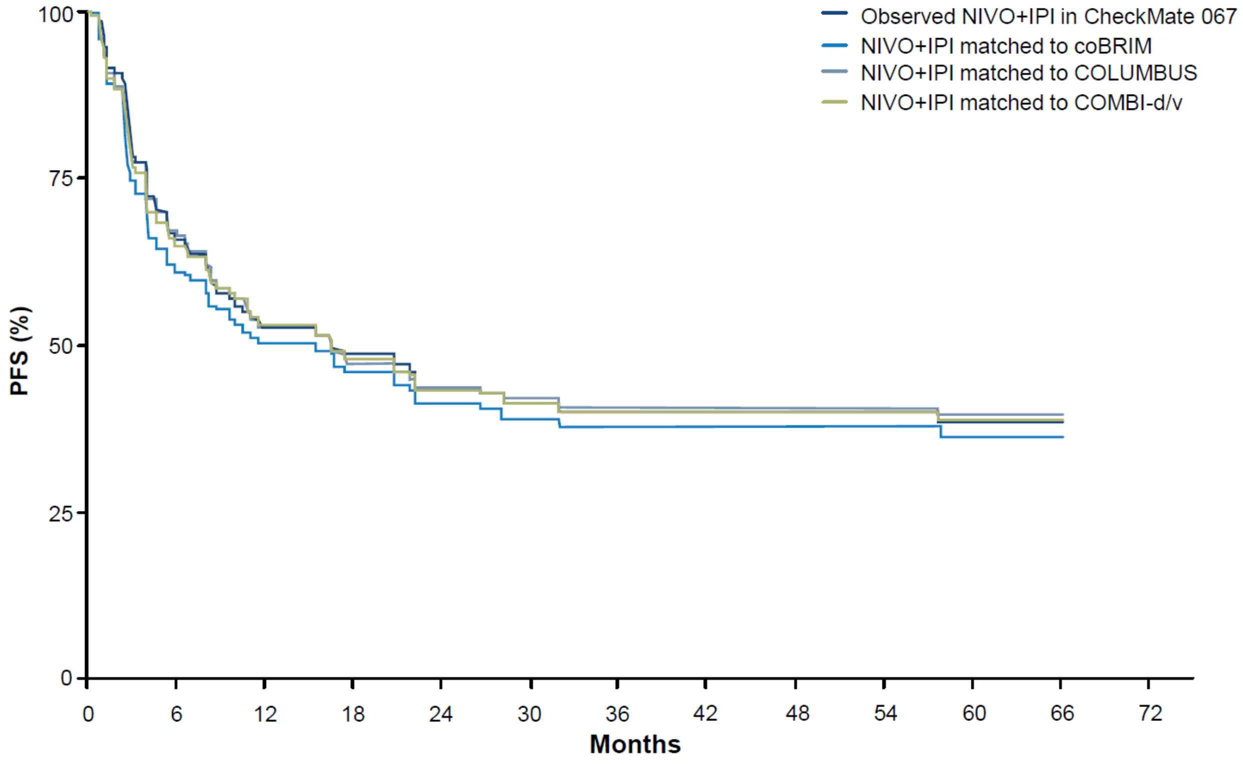

Supplement: Supplementary Material [file mmc1.pdf]
